# Alcohol use, viral hepatitis and liver fibrosis among HIV-positive persons in West Africa: a cross-sectional study

**DOI:** 10.7448/IAS.20.1.21424

**Published:** 2017-02-17

**Authors:** Antoine Jaquet, Gilles Wandeler, Marcellin Nouaman, Didier K Ekouevi, Judicaël Tine, Akouda Patassi, Patrick A Coffie, Aristophane Tanon, Moussa Seydi, Alain Attia, François Dabis

**Affiliations:** ^a^ INSERM U1219 Bordeaux Population Health Research, ISPED, Université de Bordeaux, Bordeaux, France; ^b^ Service de maladies infectieuses et tropicales, CRCF, CHU de Fann, Dakar, Sénégal; ^c^ Department of Infectious Diseases, University Hospital Bern, Bern, Switzerland; ^d^ Programme PACCI, CHU Treichville, Abidjan, Côte d’Ivoire; ^e^ Département de santé publique, Faculté des Sciences de la santé, Université de Lomé, Lomé, Togo; ^f^ Service de maladies infectieuses et tropicales, CHU Sylvanus Olympio, Lomé, Togo; ^g^ Service de maladies infectieuses et tropicales, CHU de Treichville, Abidjan, Côte d’Ivoire; ^h^ Service de hépato-gastroentérologie, CHU de Yopougon, Abidjan, Côte d’Ivoire

**Keywords:** liver fibrosis, HIV, alcohol, hepatitis B, hepatitis D, hepatitis C, Africa

## Abstract

**Introduction**: Liver fibrosis is often the first stage of liver disease in people living with HIV (PLWHIV) in industrialized countries. However, little is known about liver fibrosis and its correlates among PLWHIV in sub-Saharan Africa.

**Methods**: The study was undertaken in three HIV referral clinics in Côte d’Ivoire, Senegal and Togo. Enrolled PLWHIV underwent a non-invasive assessment of liver fibrosis combining liver stiffness measure (LSM) with transient elastography and the aspartate aminotransferase-to-platelet ratio index (APRI). Significant liver fibrosis was defined as LSM ≥7.1 kPa. Patients were screened for alcohol use (alcohol use disorder identification test (AUDIT)-C questionnaire), hepatitis B virus (HBV) antigen, hepatitis Delta virus (HDV) antibody and anti-hepatitis C (HCV) antibody. A logistic regression model was used to identify the factors associated with significant liver fibrosis.

**Results**: A total of 807 PLWHIV were screened at a median age of 43 years (interquartile range (IQR): 36–50). Their median CD4 count was 393 cells/mm^3^ (IQR: 234–563) and 682 (84.5%) were on antiretroviral therapy (ART). The prevalence of significant fibrosis was 5.3% (3.8–6.7). Infections with HBV and HCV were identified in 74 (9.2%) and nine (1.1%) participants. Main factors associated with liver fibrosis were alcohol use (AUDIT-C >6): (odds ratio (OR) = 4.0, confidence interval (CI): 1.2–14.0), (Ref. AUDIT-C <4) and HBV infection (OR = 2.9, CI: 1.2–7.2). Of the 74 patients positively screened for HBV, 50.0% were on a tenofovir-based ART regimen. Overall, 10% of HIV/HBV coinfected patients were detected with a positive HDV antibody with a higher prevalence in patients with a significant liver fibrosis (43.0%) compared to others (6.3%) (*p* = 0.01).

**Conclusions**: Considering the WHO recommendations to screen for HBV infection and treat co-infected patients with tenofovir-based ART, screening of alcohol use and brief interventions to prevent alcohol abuse should be implemented in West Africa, especially in HBV/HIV co-infected patients.

## Introduction

Liver diseases now represent one of the leading causes of mortality in people living with HIV (PLWHIV) in industrialized countries [1,2]. Of these liver-related deaths, over 60% are attributable to hepatitis C virus (HCV) [3]. According to the 2013 global burden of disease estimates, sub-Saharan Africa harbours a particularly high level of liver-related mortality through cirrhosis and hepatocellular carcinoma irrespective of HIV status [4]. Chronic infection with hepatitis B virus (HBV) represents the main etiologic factor of liver-related mortality on this continent and is endemic especially in West Africa [5]. However, one-third of cirrhosis deaths remain of unknown aetiology [4]. Liver fibrosis is often a preliminary stage preceding liver diseases. In this context, a better documentation of the burden of liver fibrosis and associated factors is now needed.

In 2015, the worldwide estimated number of PLWHIV was 36.7 million and 70% of them were living in sub-Saharan Africa [6]. With the expanding access to efficient antiretroviral therapy (ART) in sub-Saharan Africa, PLWHIV experienced a prolonged life expectancy and are now entering into a long-term life plan with co-morbidities including liver diseases [7,8]. A previous report have identified PLWHIV as vulnerable to liver fibrosis in a context of no or limited access to ART in Uganda [9]. The association between HBV infection and liver fibrosis has been previously reported among PLWHIV in Nigeria [10]. Aside HBV infection, there are limited information on other risk factors of liver fibrosis among African PLWHIV. Indeed, the respective role of HCV infection and alcohol use has been understudied so far. Alcohol use was identified as negatively influencing adherence to ART in West Africa but its impact on the occurrence of liver disease in this particular context is less known [11].

Our aim was to estimate de prevalence of liver fibrosis and investigate associated factors among PLWHIV in care in West Africa.

## Methods

### Study population

HIV-positive adults (≥18 years) currently in care were recruited during a three-month period in three infectious disease units from referral hospitals in Abidjan (Côte d’Ivoire), Dakar (Senegal) and Lome (Togo). Facing the high number of patients attending the clinics, we were unable to guarantee an exhaustive enrolment procedure. Indeed, between 200 and 500 patients per week were seen in consultation in each participating sites. A sampling procedure was performed to select a representative sample of patients attending the HIV clinics during the study period. The recruitment was ensured between two or three days per week according to operator’s availability. On recruitment days, 10–15 patients could be included. Systematic sampling, a form of one-stage cluster sampling, was applied on a daily basis. The sampling frame was the list of patients planned to attend the centre on the day of inclusion. Considering a sampling interval *k* = number of participants to be included/number of people listed to attend, monitors were asked to select among the first *k* patients on this list and then consecutively include the following patients by applying this predetermined sampling interval. If the selected person refused, participation was proposed to the next person on the waiting list. Patients were included after approving the study. They were then administered a standardized case report form in order to collect their sociodemographic characteristics (gender, age, place of living) as well as their addictive behaviours including alcohol and tobacco use.

Alcohol use during the last 12 months was measured using the short version of the alcohol use disorder identification test (AUDIT-C); a three-item questionnaire, each of them scored from 0 to 4 points, giving a maximum total score of 12 points. This test has been specifically designed to identify individuals for whom the use of alcohol places them at risk of developing alcohol problems or who are experiencing such problems. A score ≥4 in men and ≥3 in women was estimated to have 86% sensitivity and 72% specificity for risky drinking and/or active alcohol abuse or dependence [12,13]. Although this screening tool was validated in population from North America, a previous report from sub-Saharan Africa showed that brief versions of the AUDIT may be appropriately used for the screening of excessive alcohol use in PLWHIV [14]. An additional threshold ≥6 was also considered in order to target participant with heavy declared alcohol use. Although only studied in men, this conservative cutoff was associated with a significantly increased risk of liver disease, upper gastrointestinal bleeding or pancreatitis [15]. According to their AUDIT-C score, patients were thus classified as hazardous drinkers (AUDIT-C ≥4 in men, ≥3 in women) and heavy drinkers (AUDIT-C ≥6).

Self-report of tobacco use allowed the classification of the respondents as non-users, past-users or current users. The duration of tobacco uses as well as the average number of cigarettes smoked on a typical day was also reported for current and past tobacco users. Height and weight were systematically measured and reported on the case report form in order to compute the body mass index (BMI) = weight (kg)/(height (m))^2^. A cutoff of ≥30 was used to define obesity.

Information related to the characteristics of HIV infection including CD4 cell count (last known measure and measure at first entry into HIV care), the use of ART, current and past history of ART regimen were extracted from patient medical records.

Once the questionnaire was administered, all included patients underwent a blood sample collection and a measure of liver stiffness measure (LSM) through transient elastography (see later) on the same day.

### Laboratory measurements

Viral hepatitis co-infections were assessed using rapid diagnostic tests on patient’s blood samples: Determine® (Alere, Waltham, MA, United States of America (USA)) for HBs antigen and Oraquick® (Orasure, Bethlehem, PA, USA) for anti-HCV antibodies as these tests have shown relatively good diagnostic accuracy [16,17]. Aspartate aminotransferase (AST), alanine aminotransferase (ALT) and platelet count were measured for all participants in order to compute the APRI score = defined as ((AST*100)/upper limit of normal)/platelets (10^9^/l).

Those who were positive for the HBV test underwent viral load quantification by polymerase chain reaction (PCR) using the ABBOTT M2000RT (Abbot®) kit in Togo and the COBAS(R) AmpliPrep/COBAS(R) TaqMan(R) v2.0 (Roche®) kit in Senegal and Cote d’Ivoire. Results were reported in international units (UI) per millilitre. A threshold of 2000 UI/ml was considered for clinically significant viral load replication as suggested in the recent European guidelines for the management of chronic HBV infection [18]. Total hepatitis Delta virus (HDV) Ab were detected with ETI-AB-DELTA-2 (DiaSorin S.A., Antony, France). Frozen plasma samples of patients screened with a positive HCV test were centralized in the virology unit in Dakar for HCV viral load quantification by PCR using the COBAS(R) AmpliPrep/COBAS(R) TaqMan(R) v2.0 (Roche®) kit. Samples with detectable HCV viral load were tested for HCV genotype using an in-house assay. All commercial laboratory tests were performed according to the manufacturer’s specifications.

### Transient elastography

All participants were assessed for liver fibrosis using a portable transient elastography device (Fibroscan E401®, Echosens, Paris) with an M probe. A single device was made available for a three-month period in each participating ward. A maximum of two operators were specifically trained to perform the examination at each site. All operators followed the online formal training and certification from the manufacturer. Prior to the study inclusions, they all went through a practical training session with at least 50 transient elastography performed, supervised by an experienced hepatologist before being able to include patients. The median value of 10 successive validated LSM was used to represent liver stiffness. To be considered reliable, the examination must include at least 10 measurements with an interquartile range (IQR) ≤30% of the median value (IQR/Median LSM ≤30%). Transient elastography measures were considered very reliable if the IQR/Median LSM was <0.1 and reliable if IQR/Median LSM was between 0.1 and 0.3 [19]. Participants for who these criteria were not achieved after several attempts were excluded from the present analysis.

### Statistical analysis

Liver fibrosis was assessed using the combination of the following two non-invasive tests; transient elastography and APRI score. The low cutoff value of <0.5 points with the APRI score was applied to define the absence of significant liver fibrosis. Using this threshold, the APRI score has shown a relatively good negative predictive value (80% (95% confidence interval (CI): 76–84]), based on a 49% prevalence of ≥F2 stage using the METAVIR score [20]. Transient elastography was then used with a cutoff value of LSM ≥7.1 kPa to define the presence of a significant liver fibrosis and ≥14.5 kPa for cirrhosis. These thresholds for LSM were reported in a recent meta-analysis as the median optimal cutoff points to define a significant liver fibrosis that approximates a ≥F2 METAVIR score (and ≥F4 METAVIR score for cirrhosis) among populations presenting with various etiological factors for liver fibrosis [21].

Characteristics of participants with and without severe significant fibrosis were compared using Pearson’s *δ* ^2^ test or Fisher’s exact test when appropriate for categorical variables and Kruskall-Wallis test for continuous variables. A multivariable logistic regression using the Firth’s penalized likelihood method for rare events was used to assess factors associated with significant fibrosis [22]. A stepwise descending procedure was applied to derive the model that best predicted the presence of severe liver fibrosis. The goodness of fit of the model was assessed using the Akaike Information Criterion (AIC), a lower value of the AIC suggesting a better prediction of the model. All relevant potential confounders were included in the initial multivariate model. Proportions and odds ratio (OR) estimates were reported with their 95% CI. All statistical analyses were performed using SAS software 9.2 (SAS Institute Inc., NC, USA).

### Ethical considerations

All participants were informed about potential risks and benefits related to their study participation. Participants gave their signed informed consent before being included. The present study has been approved by the national ethic committees of Côte d’Ivoire (“Comité National d’Ethique pour la Recherche en Côte d’Ivoire”, approval number: 036/MSLS/CNER-dkn), Senegal (“Comité National d’Ethique pour la Recherche en Santé au Senegal”, approval number: 3226/MJ/DAP/SMS) and Togo (“Comite de Bioéthique pour la Recherche en Santé du Togo”, approval number: 004/2013).

## Results

Of the 856 participants solicited, 20 (2.4%) refused to participate. Among those who agreed to be screened for liver fibrosis, 29 (3.5%) were excluded from the present analysis for the following reasons: unreliable LSM (*n* = 20), indeterminate/unknown HBV, HCV or HIV status (*n* = 4) and age<18 years (*n* = 5).

A total of 807 HIV-positive patients were finally included in the analysis. Their median age was 43 years (interquartile range (IQR): 37–50); their median time since first follow-up for a positive HIV serology was four years (IQR: 2–8) and 682 (84.5%) of these PLWHIV were taking ART for a median duration of four years (IQR: 2–7) (Table 1).

**Table 1.** Characteristics of HIV-infected patients according to the presence of a significant liver fibrosis assessed by non-invasive markers (*n* = 807)

	No significant liver fibrosis(n=764)	Significant liver fibrosis(n=43)	p	Total(N=807)
Age (median, [IQR])	43 [37 – 50]	45 [40 - 51]	0.26	43 [37 – 50]
Gender, n (%)			<0.01	
Women	548 (71.7)	22 (51.2)		570 (70.6)
Men	216 (28.3)	21 (48.8)		237 (29.4)
Body mass index^a^			0.68	
<30	697 (91.2)	40 (93.0)		737 (91.3)
≥30	67 (8.8)	3 (7.0)		70 (8.7)
Referral hospital			<0.01	
Dakar (Senegal)	208	3 (6.7)		211 (26.1)
Lome (Togo)	228	20 (46.5)		248 (30.7)
Abidjan (Côte d’Ivoire)	328	20 (46.5)		348 (43.2)
AUDIT-C^b^ score, n (%)			<0.0001	
< 4^c^	698 (91.4)	33 (76.7)		731 (90.6)
≥ 4 - 5	43 (5.6)	1 (2.3)		44 (5.4)
≥ 6	23 (3.0)	9 (21.0)		32 (4.0)
Tobacco use			0.38	
No	643 (84.1)	34 (79.1)		677 (83.9)
Current/past	121 (15.9)	9 (20.9)		130 (16.1)
Antigen HBs, n (%)			0.10	
Negative	697 (91.2)	36 (83.7)		733 (90.8)
Positive	67 (8.8)	7 (16.3)		74 (9.2)
Antibody anti-HCV, n (%)			0.02	
Negative	757 (99.1)	41 (95.4)		798 (98.9)
Positive	7 (0.9)	2 (4.6)		9 (1.1)
Last known CD4 count (median, [IQR]), cells/mm^3^	388 [231 – 550]	321 [222 – 499]	0.29	384 [231 – 546]
First known CD4 count (median, [IQR]), cells/mm^3^	174 [69 – 299]	225 [134 – 344]	0.26	177 [69 – 300]
Antiretroviral use, years			0.04	
≤1	170 (22.2)	16 (37.2)		186 (23.0)
>1 - 5	288 (37.7)	16 (37.2)		304 (37.7)
>5	306 (40.1)	11 (25.6)		317 (39.3)
Antiretroviral regimen			0.36	
Not treated	115 (15.1)	10 (23.2)		125 (15.5)
AZT/3TC or FTC/NVP	173 (22.6)	12 (27.9)		185 (22.9)
TDF/3TC or FTC/EFV	163 (21.3)	9 (20.9)		172 (21.3)
AZT/3TC or FTC/EFV	119 (15.6)	3 (7.1)		122 (15.1)
PI-based regimen	148 (19.4)	5 (11.6)		153 (19.0)
Others	46 (6.0)	4 (9.3)		50 (6.2)

^a^Body mass index  (BMI) = weight (kg)/height^2^ (m), a BMI ≥30 is usually retain to define obesity. ^b^Declared alcohol use during the last 12 month assessed with the short version of the alcohol use disorders identification test (AUDIT). ^c^<3 for women. HIV: human immunodeficiency virus; HBV: hepatitis B virus; HCV: Hepatitis C virus; IQR: inter quartile range; AZT: zidovudine; 3TC: lamivudine; FTC: emtricitabine; NVP: nevirapine; TDF: tenofovir; EFV: efavirenz; PI: protease inhibitors.

Based on transient elastography measures; 127 (15.7%) patients were identified with a LSM≥7.1 kPa. The agreement rate between the APRI score and transient elastography was 74.0% (*p* <10^–4^), with a Kappa coefficient of 0.13. A total of 43 patients presented an APRI score ≥0.5 associated with a LSM ≥7.1 kPa providing an overall prevalence of significant fibrosis of 5.3% (95% CI: 3.8–6.7). Three patients (0.4%) presented with an LSM ≥14.5 kPa suggestive of cirrhosis, all had an APRI score ≥0.5.

Current and hazardous alcohol uses were reported in 271 (33.6%) and 78 (9.4%) of HIV-positive patients, respectively. Marked differences were observed by country with 12.5% and 12.1% of hazardous drinkers in Togo and Côte d’Ivoire versus 1.4% in Senegal (*p* <0.0001). Men were also more likely to consume alcohol with 17.3% of hazardous drinkers versus 6.1% in women (*p* <0.0001). A current or past history of tobacco use was reported by 130 (16.1%) of HIV-positive patients with a median number of 10 cigarettes (IQR: 4–15) smoked on a typical day and a median duration of smoking of 15 years (IQR: 7–24).

A total of 74 (9.2%) patients were HBV/HIV co-infected with significant differences according to clinic: 12.3% in Senegal, 10.1% in Abidjan and 5.2% in Togo (*p* = 0.02). The main characteristics of these HBV/HIV co-infected patients are presented in Table 2. The prevalence of liver fibrosis in this sub-group was 9.5% (95% CI: 6.7–16.1). Liver fibrosis was significantly more frequent in HBV-infected patients declaring a current alcohol use (21.7%) compared to no alcohol users (3.9%) (*p* = 0.01). A high ALT value (>40 IU/ml) was reported in 16 (21.6%) of these HBV/HIV co-infected patients, with more frequent significant liver fibrosis (25.0%) compared to patients with ALT<40 UI/ml (5.2%) (*p* = 0.03).

**Table 2.** Characteristics of HBV/HIV co-infected patients according to the presence of a significant liver fibrosis assessed by non-invasive markers (*n* = 74)

	No significant liver fibrosis(n=67)	Significant liver fibrosis(n=7)	p^a^	Total(n=74)
Age (median, [IQR])	41 [35 – 48]	42 [34 – 57]	0.83	41 [35 – 48]
Gender			0.18	
Women	49 (73.1)	3 (43.0)		52 (70.3)
Men	18 (26.9)	4 (57.0)		22 (29.7)
Referral hospital			0.04	
Dakar (Senegal)	26 (38.8)	0 (0.0)		26 (35.1)
Lome (Togo)	10 (14.9)	3 (43.0)		13 (17.6)
Abidjan (Côte d’Ivoire)	31 (46.3)	4 (57.0)		35 (47.3)
AUDIT-C^b^ score, n (%)			0.01	
< 4^c^	49 (73.1)	2 (28.6)		51 (70.0)
≥ 4-5	15 (22.4)	3 (42.9)		18 (24.3)
≥ 6	3 (4.5)	2 (28.6)		5 (6.7)
AST (median, [IQR])	26 [22 – 40]	39 [34 – 78]	0.01	27 [22 – 40]
ALT (median, [IQR])	22 [17 – 34]	52 [36 – 77]	0.002	24 [18 – 37]
HBV viral load (IU/L)			0.58	
<20 (undetectable)	42 (62.7)	6 (85.7)		48 (64.9)
≥20 – 2 000	17 (25.4)	1 (14.3)		18 (24.3)
≥2 000	8 (11.9)	0 (0.0)		8 (10.8)
HDV Antibody^d^			0.02	
Negative	59 (93.6)	4 (57.0)		63 (90.0)
Positive	4 (6.4)	3 (43.0)		7 (10.0)
Last known CD4 count	380 [236-487]	335 [245-425]	0.62	376 [243 – 482]
median, [IQR], cells/mm^3^				
ART regimen			0.74	
Lamivudine-based	23 (34.3)	2 (28.6)		25 (33.8)
Lamivudine+Tenofovir-based	34 (50.7)	3 (42.8)		37 (50.0)
Other, No ART	10 (14.9)	2 (28.6)		12 (16.2)
Antiretroviral use, years			0.92	
≤ 1	16 (23.9)	2 (28.6)		18 (24.3)
>1 – 5	27 (40.3)	3 (42.8)		30 (40.5)
> 5	24 (35.8)	2 (28.6)		26 (35.2)

^a^Fisher’s exact test for categorical variables or Kruskall-Wallis test for continuous variables. ^b^Declared alcohol use during the last 12 month assessed with the short version of the alcohol use disorders identification test (AUDIT). ^c^<3 for women. ^d^Only 70 of the 74 HIV/HBV co-infected patients were effectively tested for total HDV antibody.

Of the 74 HBV/HIV co-infected patients, 12 (16.2%) were not treated with ART active against HBV, 25 (33.8%) were on a lamivudine-based regimen and 37 (50.0%) were on lamivudine associated with tenofovir. The use of tenofovir in HBV/HIV co-infected patients varied according to location: 54.3% in Abidjan, 61.5% in Dakar and 15.4% in Lome (*p* = 0.02).

A detectable HBV viral load was reported in 26 (35.1%) HBV/HIV co-infected patients and eight (10.8%) presented a significant HBV replication level (>2000 IU/ml). Patients exposed to lamivudine and/or tenofovir-based ART regimens were more likely to present an undetectable HBV replication (29.0%) compared to others (66.7%) (*p* = 0.02). In the 62 HBV/HIV co-infected patients exposed to lamivudine, a significant HBV replication was reported in 3 (12.0%) patients on lamivudine and 3 (8.0%) patients on lamivudine associated with tenofovir (*p* = 0.5). No significant association was reported between the presence of a detectable HBV viral load and the presence of significant liver fibrosis (*p* = 0.76). Of the 70 HBV/HIV co-infected patients who were tested for HDV antibody, seven patients (10%) were found to be HDV-positive, with marked differences according to country; 23% in Togo, 11% in Côte d’Ivoire and none in Senegal (*p* = 0.05). A significant liver fibrosis was reported in 43% of HDV-positive patients versus 6.3% in HDV-negative patients (*p* = 0.01).

A total of nine patients were identified with a positive HCV infection based on the Oraquick® rapid diagnostic test, providing an estimated HCV prevalence of 1.1% (95% CI: 0.4–1.8). Of these nine HCV/HIV co-infected patients, five (2.0%) were from Lome, three (1.4%) from Dakar and one (0.3%) form Abidjan. Six had a detectable HCV viral load with a median value of 6.1 (IQR: 5.5–6.3) log UI/ml. Of the six patients with a detectable HCV viral load, three of these viruses were genotype 2, one genotype 1 and two could not be typed. One patient presented with a triple infection HCV/HBV and HIV.

In a multivariate analysis, factors associated with a significant liver fibrosis were a positive HBs antigen (OR = 2.5; 95% CI: 1.1–6.1), heavy alcohol use defined by an AUDIT score ≥6 (OR = 4.3; 95% CI: 1.7–10.8] (ref AUDIT score <4) and participating sites: Lome: (OR = 4.6 (95% CI: 1.4–15.2)), Abidjan (OR = 3.6 (95% CI: 1.2–11.3)) (reference: Dakar) (Table 3). Obesity was not associated with significant liver fibrosis (OR = 0.9 (95% CI: 0.3–2.7)). Table 3 presents covariates included in the final logistic model with their unadjusted and adjusted OR and respective 95% CI. A CD4 count measurement <200 cells/mm^3^, at last follow-up visit or at first entry into care, were not associated with liver fibrosis with OR of 0.9 (95% CI: 0.4–2.0) and 0.6 (95% CI: 0.3–1.8), respectively. These covariates were thus not retained in the final multivariate model.

**Table 3.** Factors associated with significant liver fibrosis in HIV-infected patients attending referral HIV clinics in Abidjan, Dakar and Lome, West Africa (*n* = 807)

	Univariate analysis			Multivariable analysis	
	*n*/*N*	OR (95% CI)	p	OR (95% CI)	p
Age			0.15		0.11
≤40 years >40 years	12/313 31/494	1 1.6 (0.8–3.2)		1 1.8 (0.9–3.6)	
Gender			<0.01		0.07
Women Men	22/570 21/237	1 2.4 (1.3–4.5)		1 1.8 (0.9–3.5)	
Referral hospital			0.02		0.04
Dakar, Senegal Abidjan, Côte d’Ivoire Lome, Togo	3/211 20/348 20/248	1 3.7 (1.2–11.7) 5.3 (1.7–16.9)		1 3.6 (1.2–11.3) 4.6 (1.4–15.2)	
Alcohol use,			<0.0001		<0.01
AUDIT-c score^a^ <4^b^ ≥4–5 ≥6	25/536 11/237 7/34	1 0.7 (0.1–3.9) 8.4 (3.6–19.5)		1 0.5 (0.1–2.5) 4.3 (1.7–10.8)	
Antigen HBs^c^			0.07		0.04
Negative Positive	36/733 7/74	1 2.1 (0.9–4.9)		1 2.5 (1.1–6.1)	
Anti-HCV antibody^d^			0.02		0.11
Negative Positive	41/798 2/9	1 6.1 (1.3–28.4)		1 4.8 (0.8–27.4)	
Antiretroviral use			0.05		0.21
≤1 >1–5 >5	16/186 16/304 11/317	1 0.6 (0.3–1.2) 0.4 (0.2–0.8)		1 0.7 (0.3–1.5) 0.5 (0.2–1.1)	

*n*/*N*: number of HIV-infected patients with significant fibrosis /total number of HIV-infected patients for a specific category. ^a^Declared alcohol use during the last 12 month scored with the short version of the alcohol use disorders identification test (AUDIT-c). ^b^<3 for women. ^c^Assessed with a rapid diagnostic test: Determine® (Alere, Waltham, MA, USA). ^d^Assessed with a rapid diagnostic test: Oraquick® (Orasure, Bethlehem, PA, USA). HIV: human immunodeficiency syndrome; HCV: hepatitis C virus; OR: odd ratio; CI: confidence interval.

## Discussion

The prevalence of liver fibrosis in our HIV-positive population was relatively low compared to previous estimates from sub-Saharan Africa. In Nigeria, Hawkins et al reported a prevalence of severe liver fibrosis of 4.7% in HIV mono-infected patients and 22.6% in HBV/HIV co-infected patients relying solely on transient elastography and using a LSM cutoff ≥9.3 kPa [10]. Using the same cutoff, another study in rural Uganda reported a prevalence of 17% of severe liver fibrosis in PLWHIV [9]. Nevertheless, these previous findings were reported in a context of limited or no access to ART. Therefore, ART through HIV viral suppression, might have directly impacted on liver fibrosis as suggested by a recent cohort study conducted in northern America that linked HIV replication with the incidence of liver fibrosis [23]. Although transient elastography is a technique rapidly mastered and easy to reproduce, it remains an operator-dependent procedure. An inadequate use of the device can lead to the overestimation of LSM. The use of the APRI score to withdrawal patients with false positive transient elastography measures partly explained the lower prevalence of liver fibrosis reported in our study. Recent WHO guidelines for HBV prevention and treatment promote the use of simple non-invasive biomarkers such as the APRI score for the follow-up of HBV-infected patients in resource-limited settings. However, according to a recent accuracy study using liver biopsy as the gold standard in patients with chronic HBV infection, the use of the APRI score for the assessment of liver fibrosis stage performed poorly in identifying patients with significant fibrosis, especially if already on antiviral treatment [24,25]. Therefore, the combination of non-invasive markers like we did in our study should continue to be investigated for the staging of liver fibrosis in specific populations such as HBV/HIV co-infected populations in sub-Saharan Africa.

The high prevalence of positive HBs Ag reported in this West African HIV-positive population was consistent with regional prevalence estimates of HBs Ag carriage from the general population suggesting that the natural history of HBV acquisition (essentially during early childhood) might be modestly influenced by HIV infection. Access to ART was similar in HBV/HIV co-infected patients compared to HIV mono-infected patients in these referral clinics. Only half of the HBV-infected patients were on a tenofovir-based regimen, which is now recommended as the preferred first line ART regimen according to the WHO guidelines [7]. Access to tenofovir varied greatly across these HIV clinics, with a particularly low access in Togo. Efforts are needed throughout West Africa to facilitate access to tenofovir in a context of a high HBV prevalence.

Marked differences were reported in positive HDV Ab according to country with high prevalence in Côte d’Ivoire and Togo compared to Senegal in accordance with the relatively low prevalence of HDV Ab previously reported in Dakar in HBV-infected blood donors [26]. However, there are currently limited or no data on HDV prevalence in many West African countries [27]. Larger prevalence studies are needed throughout endemic HBV settings to characterize the true burden of HDV infection. Liver fibrosis was higher among HBV-infected patients harbouring HDV antibody supporting the negative impact of HDV in HIV/HBV co-infected persons already reported in industrialized countries [28,29].

A low prevalence of HCV infection was observed in our study population, quite lower than the available regional estimates (6.7% (95% CI: 4.8–8.5)) from a recent meta-analysis conducted among HIV-positive persons in sub-Saharan Africa [30]. These previous estimates also showed a quite important heterogeneity between countries in West Africa with HCV prevalence <3% in Senegal and Cote d’Ivoire while no HCV prevalence data was available for Togo. Although limited to a selected population of HIV-positive patients attending referral hospitals, we provide here HCV prevalence estimates confirmed with HCV nucleic acid testing. These data will contribute to provide more robust prevalence estimates to inform prevention and treatment programmes against HCV infection.

The prevalence of hazardous alcohol use was high in our population, three-times higher than in our previous survey conducted five years ago in the same West African network of HIV clinics [11]. As HIV-positive patients are now on a long-term treatment plan, they might be more exposed to alcohol use and thus deserve more preventive and corrective measures. There is thus a need of longitudinal screening of alcohol use in HIV-positive people in routine care to identify potential trends in alcohol misuses that might impact the effectiveness of ART programmes in sub-Saharan Africa. Heavy alcohol use was identified as a factor strongly associated with significant liver fibrosis in our population. The etiologic role of alcohol in the development of liver fibrosis, and ultimately liver cirrhosis, is well known. However, the importance of this factor has been neglected so far in West Africa. The impact of alcohol seems to be synergic with HBV infection as HBV/HIV co-infected patients were five times more likely to present a significant liver fibrosis when declaring a current alcohol use compared to those declaring no alcohol use.

The majority of HIV-positive participants were currently on ART. Although, the duration of ART seemed to be inversely associated with significant liver fibrosis in unadjusted analysis, this association did not remain significant in the final multivariate model. There is currently no clear evidence of a protective effect of ART over time on the occurrence of liver fibrosis in HIV mono-infected patients [31,32]. Results from a recent cohort study conducted in northern America reported that a high HIV viral load was predictive of incident liver fibrosis [23]. This finding suggests that through its suppressive effect on HIV replication, ART has the potential to prevent and/or reduce liver fibrosis in HIV-positive patients. Conversely, exposure to ART drug regimens with a known hepatic toxicity could also promote liver fibrosis [33,34]. The cross-sectional nature of our study design prevents any causal relation between ART use and the occurrence of liver fibrosis. Prospective cohort studies assessing the impact of ART on liver fibrosis evolution in HIV mono-infected patients over time will be ultimately needed. Contrarily to previous studies, no association was reported between a low CD4 count and the presence of a significant liver fibrosis [23]. In our study, CD4 count measures were not considered as a time-dependent variable but was only assessed twice; at first entry into care and at last follow-up visit. This approach might have missed any association between liver fibrosis and CD4 count as we did not have information on time spent by patients in an immune-compromised state.

### Limitations

Caution must be taken in the interpretation of the prevalence of liver fibrosis. Indeed, there is a clear lack of validation studies for the chosen thresholds of the APRI score as well as transient elastography in our specific population, especially among PLWHIV without HBV infection. This absence of consensual definition is highlighted by the diversity of thresholds used in previous reports based on transient elastography in sub-Saharan Africa [9,10,35]. Although recommended by the WHO in its recent guideline on HBV care and treatment, the use of the APRI score have shown poor diagnostic performances among HBV-infected in sub-Saharan Africa. Although it performed equivalently to transient elastography for the diagnosis of a significant fibrosis (≥F2 METAVIR score) in terms of negative predictive value, it was consistently inferior in terms of positive predictive value [25,36,37]. There are various reasons for the APRI score to be falsely elevated in the context of HIV infection due to both non-liver diseases related elevation of ALT and thrombocytopenia. Excluding patients with a positive APRI score might have prevented this bias. However, the APRI score may also give rise to false negative results for significant fibrosis even at a low threshold, for example, through impaired immune responses giving rise to limited hepatic inflammation. Excluding patients with a negative APRI score might have underestimate de true prevalence of significant liver fibrosis. A significant difference was reported in prevalence of liver fibrosis according to participating clinics. This geographical variation might be related to unmeasured confounding factors. Indeed, several factors potentially influencing the occurrence of a liver fibrosis were not measured in our study such as exposure to aflatoxins or co-infection with other agents with potential hepatic tropism such as mycobacterium tuberculosis. An information bias might have occurred as transient elastography remains an operator-dependent procedure. To limit this potential effect, a maximum of two operators were allowed per site and were trained in a standardized manner. In addition, the use of the APRI score to define significant liver fibrosis showed the same tendency with higher prevalence of liver fibrosis (APRI score ≥0.5) in Lome and Cote d’Ivoire compared to Senegal. Self-reported alcohol use is subjected to underreporting of the true level of alcohol consumption. To ensure that participants freely declared their personal alcohol use, participants were informed that responses provided during the interview will be anonymously recorded. None of their responses will have any negative impact on their access to care. In addition, all clinical monitors that administered the AUDIT-C were previously trained to characterize the standard unit of alcohol intake and its various correspondences. Cutoffs used to define excessive alcohol use with AUDIT-C relied on validation studies conducted in North American populations. There is thus a need of further studies to assess these optimal cutoffs in sub-Saharan Africa. To date, there are no studies from sub-Saharan Africa demonstrating the diagnostic accuracy of the Oraquick test for the screening for HCV infection. Previous reports have point out a risk of overestimation of HCV prevalence with classical immunoassay technics potentially related to cross reaction with parasitic infections such as schistosomiasis [38,39]. However, the relatively low prevalence of HCV infection reported in our study confirmed by HCV viral quantification might limit this bias in the estimation of HCV prevalence. Finally, our study population might not be fully representative of all HIV-positive patients in care in West Africa as they were followed in referral wards with potential differences compared to patients followed in community-based HIV clinics.

## Conclusions

Heavy alcohol use was not uncommon among HIV-positive patients and identified as an important determinant of liver fibrosis in this West African population. HBV infection was also significantly associated with liver fibrosis and seems to have a synergic effect with alcohol use on the presence of liver fibrosis.

Screening of alcohol use and specific interventions to prevent alcohol abuse should be systematically proposed to HIV-positive persons in care in West Africa.

Despite the latest WHO recommendation promoting tenofovir-based first-line ART, access to this drug is still challenging in many parts of Africa. Thus, the screening of HBV infection remains a priority to ensure enhanced access to anti-HBV active drugs in HIV/HBV co-infected persons in sub-Saharan Africa. In addition, the identification of chronic Ag HBs carriers among PLWHIV is also crucial for a baseline assessment of the impact of HBV on the liver and for screening purpose to prevent hepatocellular carcinoma.
